# Sub-Inhibitory Concentrations of Ciprofloxacin Alone and Combinations with Plant-Derived Compounds against *P. aeruginosa* Biofilms and Their Effects on the Metabolomic Profile of *P. aeruginosa* Biofilms

**DOI:** 10.3390/antibiotics10040414

**Published:** 2021-04-09

**Authors:** Didem Kart, Tuba Reçber, Emirhan Nemutlu, Meral Sagiroglu

**Affiliations:** 1 Department of Pharmaceutical Microbiology, Faculty of Pharmacy, Hacettepe University, Ankara 06100, Turkey; mozalp@hacettepe.edu.tr; 2 Department of Analytical Chemistry, Faculty of Pharmacy, Hacettepe University, Ankara 06100, Turkey; tuba.recber@hacettepe.edu.tr (T.R.); enemutlu@hacettepe.edu.tr (E.N.); 3 Bioanalytic and Omics Laboratory, Faculty of Pharmacy, Hacettepe University, Ankara 06100, Turkey

**Keywords:** *P. aeruginosa*, plant-derived compounds, metabolomic, MBIC, biofilm

## Abstract

Introduction: Alternative anti-biofilm agents are needed to combat *Pseudomonas aeruginosa* infections. The mechanisms behind these new agents also need to be revealed at a molecular level. Materials and methods: The anti-biofilm effects of 10 plant-derived compounds on *P. aeruginosa* biofilms were investigated using minimum biofilm eradication concentration (MBEC) and virulence assays. The effects of ciprofloxacin and compound combinations on *P. aeruginosa* in mono and triple biofilms were compared. A metabolomic approach and qRT-PCR were applied to the biofilms treated with ciprofloxacin in combination with baicalein, esculin hydrate, curcumin, and cinnamaldehyde at sub-minimal biofilm inhibitory concentration (MBIC) concentrations to highlight the specific metabolic shifts between the biofilms and to determine the quorum sensing gene expressions, respectively. Results: The combinations of ciprofloxacin with curcumin, baicalein, esculetin, and cinnamaldehyde showed more reduced MBICs than ciprofloxacin alone. The quorum sensing genes were downregulated in the presence of curcumin and cinnamaldehyde, while upregulated in the presence of baicalein and esculin hydrate rather than for ciprofloxacin alone. The combinations exhibited different killing effects on *P. aeruginosa* in mono and triple biofilms without affecting its virulence. The findings of the decreased metabolite levels related to pyrimidine and lipopolysaccharide synthesis and to down-regulated alginate and *lasI* expressions strongly indicate the role of multifactorial mechanisms for curcumin-mediated *P. aeruginosa* growth inhibition. Conclusions: The use of curcumin, baicalein, esculetin, and cinnamaldehyde with ciprofloxacin will help fight against *P. aeruginosa* biofilms. To the best of our knowledge, this is the first study of its kind to define the effect of plant-based compounds as possible anti-biofilm agents with low MBICs for the treatment of *P. aeruginosa* biofilms through metabolomic pathways.

## 1. Introduction

Biofilm-related infections have major importance because of their clinical challenges, such as persistence and treatment approach. *Pseudomonas aeruginosa*, which is difficult to treat because of its resistance to many antibiotics and its ability to form a biofilm, is a significant pathogen that causes hospital-acquired infections [1]. *P. aeruginosa* is frequently isolated together with *Staphylococcus aureus* and *Enterococcus faecalis* from the specimens of patients with biofilm-related infections [2]. There is an urgent need to develop new antimicrobial approaches that prevent or inhibit *P. aeruginosa* biofilm formation or display a microbicidal activity on the cells in preformed biofilms [3]. Quorum sensing is a communication system in which microorganisms control their population density through small signal molecules [4]. Because of the importance of QS controlled ability for the formation and/or dispersion of *P. aeruginosa* biofilms, QS inhibitors are recommended as alternative anti-biofilm agents to antibiotics [5]. QS inhibitors can limit the development of drug resistance by applying selective pressure at concentrations below the minimum inhibitory concentration (MIC) [6]. Because of its non-toxic nature, the effect of various natural compounds on *P. aeruginosa* biofilms has been studied. Although the inhibitory effects of some natural compounds on the *P. aeruginosa* QS system are known, their effects on *P. aeruginosa* cells in polymicrobial biofilm media are still under investigation [7,8]. Investigating the effects of these natural compounds on biofilm cells with genomic and metabolomic analyzes will be effective for elucidating their anti-biofilm mechanisms.

The present study determined the minimal biofilm inhibitory concentration (MBIC) and minimum biofilm eradication concentration (MBEC) of ciprofloxacin in combination with plant-derived compounds such as curcumin, azathioprine, resveratrol, catechin hydrate, baicalin hydrate, L-canavanine, 4-nitropyridine N-oxide, p-benzoquinone, esculin hydrate, and cinnamaldehyde against *P. aeruginosa* biofilm cells. The effect of sub-MBIC concentrations of curcumin, baicalein, cinnamaldehyde, and esculin hydrate, which show the best synergistic activity with ciprofloxacin, on the QS genes and virulence characteristics of *P. aeruginosa* was investigated. To observe the antimicrobial effects of these compounds and the ciprofloxacin combinations on polymicrobial biofilms at sub-MBIC concentrations, *P. aeruginosa* cells in mono- and triple-species biofilms were separately treated with them and the results were compared. A GC-MS based metabolomic approach was used to reveal the underlying mechanisms of the anti-biofilm and anti-QS activity of curcumin, baicalein, cinnamaldehyde, and esculin hydrate.

## 2. Materials and Methods

### 2.1. Bacterial Strains and Growth Conditions

*P. aeruginosa* ATCC 47085 (PAO1), *E. faecalis* ATCC 47077, and *S. aureus* ATCC 35556 were used in the study. Fresh cultures were grown on brain heart infusion agar (BHIA; Oxoid Ltd., Hampshire, UK) at 37 °C for 24 h. The overnight cultures were prepared by subculturing bacterial strains in brain heart infusion broth (BHI; Oxoid Ltd., Hampshire, UK) and incubated at 37 °C for 24 h. For all of the experiments, the final bacterial suspensions were diluted with BHI to 10^6^ colony forming units per mL (cfu/mL).

### 2.2. Antibiotics and Plant-Derived Compounds

Curcumin, azathioprine, resveratrol, catechin hydrate, baicalin hydrate, L-canavanine, 4-nitropyridine N-oxide, p-benzoquinone, esculin hydrate, cinnamaldehyde, and ciprofloxacin were purchased from Sigma Chemical Co. (St. Louis, MO, USA). Stock solutions of ciprofloxacin and the compounds were prepared in dimethyl sulfoxide (pure DMSO), sterile water, or hydrochloric acid according to the manufacturer’s recommendations.

### 2.3. MIC, MBIC, and MBEC Assay

The MICs of ciprofloxacin and the compounds were determined using the broth microdilution method according to Clinical Laboratory Standards Institute (CLSI) guidelines [9]. *P. aeruginosa* ATCC 27853 was used as quality control for this test.

MBEC Assay^TM^ (Physiology and Genetics, P&G, Innovotech Inc., Edmonton, AB, Canada) was used to evaluate the anti-biofilm activity of the agents against *P. aeruginosa* PAO1 biofilms following the test protocol provided by the manufacturer. Briefly, 150 μL of *P. aeruginosa* suspension containing 10^6^ cfu/mL was transferred to each test well and the MBEC assay plate lids with 96 pegs were placed in the microtiter plate wells to form 24 h mature biofilms at 37 °C. After washing, the peg lids were placed into the challenge plate wells, which contained a serial two-fold dilution of each agent alone and their combinations for 24 h of incubation. In columns 1 to 11, the tested concentration ranges of ciprofloxacin and the compounds were 1–0.001 mg/L and 8.2–0.008 mg/L, respectively. After the MBIC of ciprofloxacin was determined to be 0.128 mg/L, the test concentration ranges for use in combination with the compounds were determined as 0.128–0.00128 mg/L between columns 1 to 11. Following incubation, the sonication steps were performed to transfer the biofilms on the pegs to the recovery medium, followed by overnight incubation. The optical density of each well was measured at 550 nm. MBEC is defined as the lowest concentration of a compound that prevents the regrowth of bacteria from treated-biofilms and MBIC is the minimum concentration that inhibits bio-film formation. Sub-MBIC values corresponding to 1/2× MBIC of combinations of ciprofloxacin with compounds were used in the subsequent biofilm experiments, including the virulence, gene expressions, and metabolomic analyzes.

### 2.4. Evaluation of the Antibiofilm Effects of Ciprofloxacin in Combination with the Compounds on Mono- and Triple-Species Biofilms of P. aeruginosa PAO1 and SEM Analysis

*P. aeruginosa* ATCC 47085 (PAO1) biofilms and triple-species biofilms formed by *P. aeruginosa* PAO1, *E. faecalis* ATCC 47077, and *S. aureus* ATCC 35556 were formed in 96-well microtiter plates as described by Kart et al. [10]. For triple-species biofilms, the starting inoculum of *E. faecalis* and *S. aureus* in equal volume with *P. aeruginosa* was used.

Ciprofloxacin alone and the combination of ciprofloxacin with compounds at subMBIC (1/2× MBIC) concentrations were applied on the mature mono- and triple-species biofilms. For the quantification of *P. aeruginosa* in the mono- and triple-species biofilms, the plating was performed on BHIA and Cetrimide agar, respectively. After 24 h, the colonies were counted as cfu/mL. All of the experiments were carried out at least three times.

SEM analyses were performed to visualize mono- and triple-species biofilms (Supplement Figure S1). Briefly, final suspensions of *P. aeruginosa*, *S. aureus,* and *E. faecalis* strains containing ~10^6^ CFU/mL in BHI were added alone (for *P. aeruginosa*) and in combination (1:1, vol/vol) onto the coverslips and were incubated at 37 °C for 24 h. Following the incubation time, the coverslips were washed twice with phosphate-buffered saline and fixed in a buffer containing 2% glutaraldehyde and 0.1 M cacodylate for 30 min, followed by rinsing three times for 10 min in a 0.2 M cacodylate buffer. After passing them through serial ethanol solutions, the samples were dried and then coated with gold-palladium [11].

### 2.5. Alterations in Virulence Characteristics of P. aeruginosa Biofilm Cells

#### 2.5.1. Swarming

Ciprofloxacin (0.0005, 0.001, and 0.004 mg/L), compounds (the same sub-MBIC values in the combinations) and ciprofloxacin-compound combinations (1/2× MBIC) were added to nutrient agar (8 g/L) supplemented with 5% glucose. The plates were then inoculated with 2 μL of diluted *P. aeruginosa* biofilm cultures and incubated for 16 h at 37 °C. The zone diameter on the surfaces was measured to assess the swarming activity.

#### 2.5.2. Twitching

Luria–Bertani agar plates were prepared to contain ciprofloxacin (0.0005, 0.001, and 0.004 mg/L), compounds (the same sub-MBIC values in the combinations), and ciprofloxacin–compound combinations (1/2× MBIC). The biofilm cultures of *P. aeruginosa* were stabbed from the agar surface to the bottom of the plate with a sterile needle loop. The plates were then incubated at 37 °C for 48 h. After the agar was removed, the polystyrene surface of the plates was gently washed and stained with crystal violet (0.1%, *w*/*v*). The twitching motility was assessed by measuring the diameter of the stained zone, indicating that bacteria were attached to the polystyrene surface.

#### 2.5.3. Quantification of Pyocyanin

Briefly, 1 mL of the treated (1/2× MBIC) and untreated *P. aeruginosa* biofilm cultures was extracted with 1 mL of chloroform and then re-extracted into 1 mL of 0.2 N HCl to give a pink solution. The pyocyanin absorbance of this acidic solution was measured at 520 nm [12]. The results are expressed as % reduction in pyocyanin in treated biofilm cultures compared with untreated biofilm cultures.

### 2.6. Expression of the QS-Related Genes in Biofilms

The total RNAs in biofilm cells treated with sub-MBICs (0.0005 and 0.001 mg/L) of ciprofloxacin alone and ciprofloxacin (0.0005 and 0.001 mg/L) in combination with curcumin (0.004 mg/L), baicalein (0.004 mg/L), cinnamaldehyde (0.008 mg/L), and esculetin hydrate (0.008 mg/L) were obtained by RNA Isolation Kit (Roche Life Science) according to the manufacturer’s instructions. The measurements of the mRNA levels of the QS-related genes were carried out based on our previous reference study [13]. The primers used are listed in Supplementary Table S2.

### 2.7. Metabolomic Analysis

Metabolites were extracted from the biofilm cells treated with ciprofloxacin (0.0005 and 0.001 mg/L) alone and ciprofloxacin (0.0005 and 0.001 mg/L) in combination with curcumin (0.004 mg/L), baicalein (0.004 mg/L), cinnamaldehyde (0.008 mg/L), and esculetin hydrate (0.008 mg/L). GC–MS-based measurement of the metabolites was carried out based on our previous reference study [11] (Table S1).

### 2.8. Statistical Analysis

The colony counts were recorded for each treatment as Log10. All statistical calculations were performed on the log density values. The statistical significance of the data was determined by Student’s t-test for paired samples or by the non-parametric Wilcoxon signed-rank test. A *p*-value of <0.05 was considered significant.

## 3. Results

### 3.1. Effect of the Compounds and Ciprofloxacin on the Planktonic and Biofilm Cells of P. aeruginosa

Based on the broth microdilution method, the MIC values of all compounds and antibiotics against *P. aeruginosa* are shown in Table 1. When ciprofloxacin and the compounds were administered both alone and in combination, the MBIC and MBEC values obtained against the *P. aeruginosa* biofilm cells were determined by the MBEC test. The compounds alone (8.2–0.004 mg/L) did not affect destroying cells in either biofilm. However, in the presence of the compounds, it was observed that the MBICs of ciprofloxacin were much lower compared with the MBIC of ciprofloxacin alone (Table 1). Of all the compounds in the combinations, curcumin, baicalein, esculetin hydrate, and cinnamaldehyde were the most effective ones at reducing the MBICs of ciprofloxacin. No significant difference was observed in MBEC values when ciprofloxacin was administered alone or in combination with compounds (Table 1).

### 3.2. Comparison of the Effect of Compound–Ciprofloxacin Combinations with Sub-MBIC Values on Mono- and Triple-Species Biofilms of P. aeruginosa

**Figure 1:**
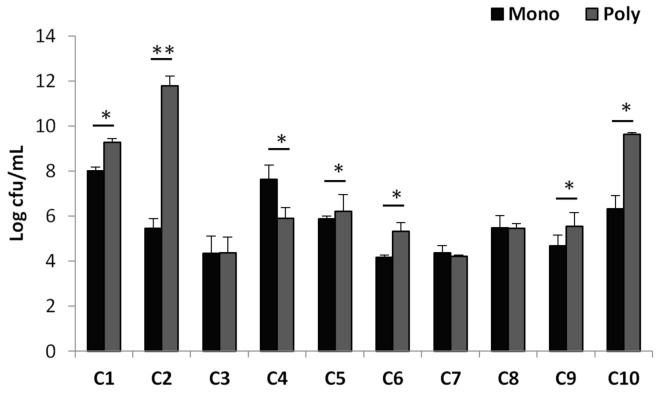
Viable cell counts of *P. aeruginosa* in mono- and triple-species biofilms. C1: Ciprofloxacin + Curcumin; C2: Ciprofloxacin + Azathioprine; C3: Ciprofloxacin + Resveratrol; C4: Ciprofloxacin + Catechin hydrate, C5: Ciprofloxacin + Baicalein, C6: Ciprofloxacin + L-canavanine, C7: Ciprofloxacin + 4-nitropyridine N-oxide; C8: Ciprofloxacin + p-benzoquinone; C9: Ciprofloxacin + Esculetin hydrate; C0: Ciprofloxacin + Cinnamaldehyde. * Significant (*p* < 0.05), ** Significant (*p* < 0.01). The results are shown as the average ± standard deviation.

Mono- and triple-species biofilm models have been developed to understand whether the efficacy of antimicrobial combinations at sub-MBIC concentrations differ when *P. aeruginosa* is in a mixed biofilm environment compared with alone. When ciprofloxacin was combined with azathioprine, an approximately 6 logarithm further reduction in the number of *P. aeruginosa* viable cells in the mono-species biofilms than that of triple-species biofilm was observed. In contrast, the viable cell counts in mono-species biofilms treated with the combination of ciprofloxacin with curcumin, catechin hydrate, baicalein, L-canavanine, esculin hydrate, and cinnamaldehyde were found to be less than the triple-species biofilms (Figure 1). No significant difference was observed in the cell numbers treated with the combination of ciprofloxacin with resveratrol, 4-nitropyridine N-oxide, and p-benzoquinone in both biofilm models (Figure 1). According to our results, combinations of antimicrobial agents may show different effects in mono- or triple-species biofilms.

### 3.3. Influence of the Compounds and Ciprofloxacin Combinations on Virulence Properties of P. aeruginosa

**Figure 2:**
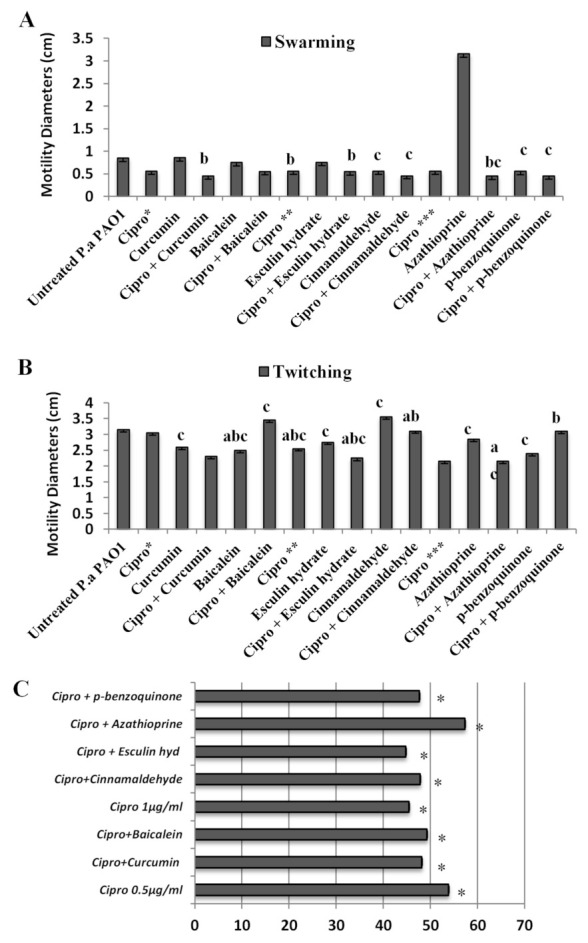
The effect of the antimicrobial agents alone and in combination on the *P. aeruginosa* biofilm cells: (**A**) twitching motilities and (**B**) swarming motilities. The measurements of the swarming and twitching activity (in centimeter) were performed on the agar plates inoculated with the cells detached from biofilms treated with antimicrobial agents. Cipro*: Ciprofloxacin at 0.0005 mg/L; Cipro**: Ciprofloxacin at 0.001 mg/L; Cipro***: Ciprofloxacin at 0.004 mg/L. (a) It is significantly different than the cells treated with ciprofloxacin (*p* < 0.05). (b) It is significantly different than the cells treated with the compounds (*p* < 0.05). (c) It is significantly different than the untreated cells (*p* < 0.05). (**C**) Quantitation of pyocyanin production in the biofilm cells treated with ciprofloxacin alone and in combination with the compounds. * Significant (*p* < 0.05). The results are expressed as % reduction.

Compared with the untreated *P. aeruginosa* biofilms, treatment with cinnamaldehyde and p-benzoquinone caused a significant inhibition in the swarming motility of bacteria, while treatment with azathioprine resulted in a significant increase in the cells motility (Figure 2A). No inhibition of swarming motility was observed in biofilm cells treated with curcumin, baicalein, and esculin hydrate compared with the control. Combinations of all compounds tested with ciprofloxacin exhibited an inhibitory effect similar to that of ciprofloxacin alone compared with untreated biofilm cultures. In addition, no additional contribution of the combinations to ciprofloxacin in inhibiting *Pseudomonas* swarming activity was observed (Figure 2A).

For the twitching activity, ciprofloxacin and all compounds except cinnamaldehyde caused statistically significant inhibition compared with the untreated control. The twitching activity of *P. aeruginosa* was inhibited after treatment with ciprofloxacin and all compounds tested separately, while in combination, only ciprofloxacin with the curcumin and esculetin hydrate compounds were effective in inhibiting the twitching motility of the cells. However, when baicalein was combined with ciprofloxacin, the motility of the cells was increased (Figure 2B).

### 3.4. The Impact of Ciprofloxacin Combined with the Compounds on QS Gene Expressions of P. aeruginosa Biofilm Cells

**Figure 3:**
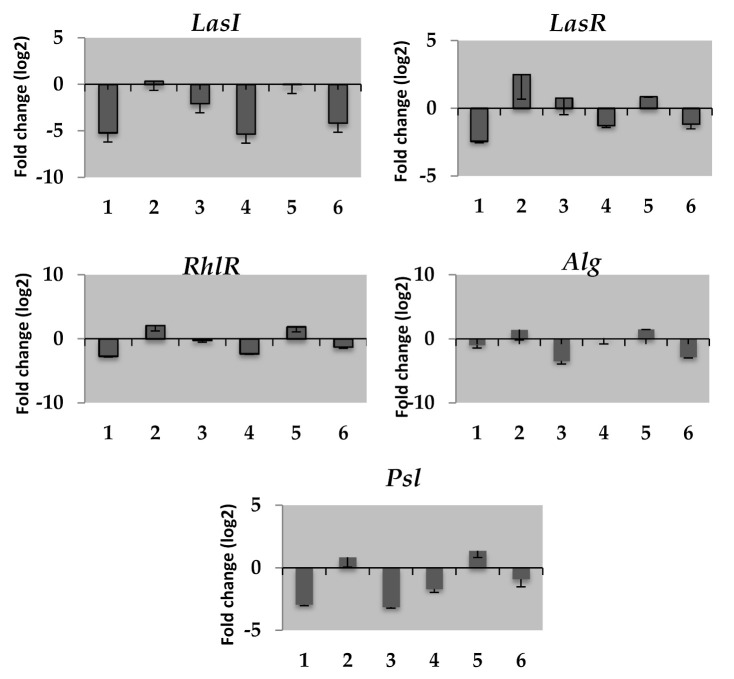
The mean relative mRNA expression levels of quorum sensing genes in *P. aeruginosa* cells treated with ciprofloxacin alone and in combination with curcumin, cinnamaldehyde, baicalein and esculetin hydrate. *Indicates statistically significant (*p* < 0.05) 1. Ciprofloxacin (0.5 µg/mL), 2. Ciprofloxacin (0.5 µg/mL) and Baicalein (4 µg/mL), 3. Ciprofloxacin (0.5 µg/mL) and Curcumin (4 µg/mL), 4. Ciprofloxacin (1 µg/mL), 5. Ciprofloxacin (1 µg/mL) and Esculetin hydrate (8 µg/mL), 6. Ciprofloxacin (1 µg/mL) + Cinnamaldehyde (8 µg/mL).

In cells treated with both ciprofloxacin alone and ciprofloxacin in combination with curcumin and cinnamaldehyde on the expression levels of *lasl*, *alg,* and *psl* genes related to QS and biofilm formation were decreased. On the contrary, increased expressions were observed in cells treated with ciprofloxacin in combination with baicalein and esculin hydrate (Figure 3).

### 3.5. Effect of Ciprofloxacin and Compounds Combinations on the Metabolic Profile of P. aeruginosa Biofilm Cells

**Figure 4:**
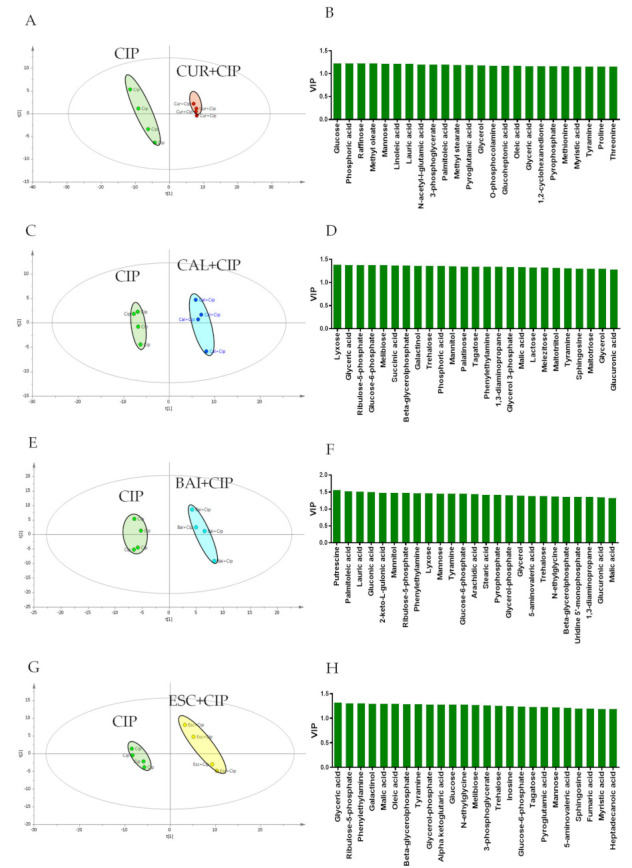
Partial Least-Squares Discriminant Analysis score chart and Variable importance charts obtained after the comparison of biofilm cells treated with ciprofloxacin alone with those treated with combinations of curcumin, cinnamaldehyde, baicalein, and esculetin hydrate. (**A**) PLS-DA score plot shows a clear separation between *P. aeruginosa* cells treated with ciprofloxacin alone and in combination with curcumin. The R2 and Q2 values for the biofilm samples were 0.999 and 0.983, respectively. (**C**) PLS-DA score plot shows a clear separation between *P. aeruginosa* cells treated with ciprofloxacin alone and in combination with cinnamaldehyde. The R2 and Q2 values for the biofilm samples were 0.998 and 0.971, respectively; (**E**) PLS-DA score plot shows a clear separation between *P. aeruginosa* cells treated with ciprofloxacin alone and in combination with baicalein. The R2 and Q2 values for the biofilm samples were 0.995 and 0.960, respectively. (**G**) PLS-DA score plot shows a clear separation between *P. aeruginosa* cells treated with ciprofloxacin alone and in combination with esculetin hydrate. The R2 and Q2 values for the biofilm samples were 0.998 and 0.990, respectively. (**B**,**D**,**F**,**H**) VIP charts of metabolites that are effective in separating the groups.

After treatment with both sub-MBICs of ciprofloxacin and combinations of ciprofloxacin with curcumin, baicalein, cinnamaldehyde, and esculetin hydrate, the changed metabolite profiles in the biofilm cells are shown in Table 2 and Figure 4. Many metabolic pathways such as carbohydrates, amino acids, nitrogen, amines, lipids, and nucleotides were reduced following the addition of curcumin compared with ciprofloxacin alone. Compared with ciprofloxacin alone, statistically significant changes in the metabolites involved in the carbohydrates, amines, and nucleotide pathways were observed in the cells treated with ciprofloxacin–baicalein and ciprofloxacin–esculetin hydrate combinations (Table 3). Compared with baicalein and esculetin hydrate, cinnamaldehyde had different metabolite effects on the amine and pyrimidine pathways. Decreased uracil, uridine monophosphate (UMP), and ribulose-5-phosphate levels were observed in the cells treated with combinations of ciprofloxacin with curcumin and cinnamaldehyde, while increased levels of uracil and UMP were observed in cells treated with combinations of ciprofloxacin with baicalein and esculin hydrate (Table 2 and Table 3).

The multivariate statistical analysis of GC–MS metabolomics results was performed using PCA (Supplement Figure S1) and PLS-DA methods. PLS-DA analysis was performed to further investigate the altered metabolite profile in cells when ciprofloxacin was administered in combination with curcumin, cinnamaldehyde, baicalein, and esculin hydrate, respectively, compared with ciprofloxacin alone (Table 2). Firstly, the statistical goodness and robustness of the models were evaluated using R2 (the fraction of variance explained by a component) and Q2 (the fraction of the total variation predicted by a component) values, respectively. The R2 and Q2 values for the models were higher than 0.9 for both, indicating the validity of the methods and showing that the models were stable and used the settings for the variables to calculate the predictions. The scores plots showed that the metabolomics profile of ciprofloxacin and ciprofloxacin plus curcumin, cinnamaldehyde, baicalein, and esculetin hydrate were significantly different from each other (Figure 4A,C,E,G). The VIP graphs are shown in Figure 4B,D,F,G.

## 4. Discussion

A decrease in tolerance to antimicrobials can be observed in *P. aeruginosa* cells living in biofilms compared with planktonic forms. Natural compounds such as cinnamaldehyde, curcumin, esculetin hydrate, and baicalein can be considered as alternative antimicrobial agents that are not affected by drug resistance developed by bacteria. The inhibition of the QS network is a potential strategy for controlling infections caused by *P. aeruginosa*. The impacts of various natural and synthetic compounds on the QS system of bacteria have been investigated [14,15,16]. Investigating the effects of sub-MIC concentrations of agents that can be alternatives to antibiotics for biofilm formation is particularly important for combating both drug resistance and biofilm-related infections, as they are less likely to apply selective pressure for resistance development [6]. In recent years, anti-biofilm effect studies involving drug combinations have shown that the effects of the combinations are higher than for the single agents [17,18]. It was reported in a study that *S. aureus* and *C. albicans* were resistant to curcumin and imidazole both in mono and dual biofilms, but when these agents were used together, there was a significant decrease in the number of living cells in the biofilms [18]. In the study by Bahari et al., it was shown that the combination of azithromycin and gentamicin with curcumin significantly reduced the MIC values of the antibiotics, the clumping and twitching motility of *P. aeruginosa*, and the formation of biofilms [15]. Sub-MIC concentrations of cinnamaldehyde were shown to be able to decrease the formation of biofilms by *S. epidermidis*, MRSA, and *E. coli* [14,19,20]. The antibacterial activity of catechin hydrate, a flavonoid found in tea, wine, some fruits, vegetables, and chocolate, against *S. aureus* clinical strains and the synergistic effect of catechin hydrate with erythromycin and clindamycin were reported in a study [21]. In addition, it has been found to inhibit the biosynthesis of QS-associated virulence factors in *P. aeruginosa* [22].

In our study, we determined the MBIC and MBEC values by examining the anti-biofilm effect of 10 potential QS inhibitor compounds and ciprofloxacin against *P. aeruginosa* biofilm cells, both alone and in combination. Similar to the literature, our results showed that ciprofloxacin and compound combinations were effective at inhibiting *P. aeruginosa* at lower concentrations compared with ciprofloxacin alone. No effect for the combinations on reducing the MBEC of ciprofloxacin was observed, although the most reductions in the MBICs of ciprofloxacin were determined when in combination with curcumin, baicalein, esculetin hydrate, and cinnamaldehyde. These combinations with low MBICs may be significant in the treatment of biofilm-associated *P. aeruginosa* infections.

Ciprofloxacin and the compounds alone showed an inhibitory effect on both the motility and pyocyanin synthesis of *P. aeruginosa*. However, except for the effects of combinations of ciprofloxacin with curcumin and esculetin hydrate on the cells’ twitching activity, a synergistic effect increasing the inhibition was not observed for the other combinations.

Antimicrobial treatment efficacy was less successful in dual- or multiple-species biofilms in vitro than biofilms alone [23,24,25]. As biofilm infections are caused by microbial cells consisting of more than one species in the clinic, we compared the effects of the agent combinations both in *P. aeruginosa* alone and in triple-species biofilms. Azathioprine in combination with ciprofloxacin was determined to be more effective at killing *P. aeruginosa* in mono-species biofilms compared with the triple-species biofilm formed by *P. aeruginosa*, *S. aureus,* and *E. facealis*. However, contrasting results were obtained for combinations of ciprofloxacin with curcumin, catechin hydrate, baicalein, L-canavanine, esculin hydrate, and cinnamaldehyde. These results show that the presence of a polymicrobial biofilm environment should also be taken into account for treatment strategies.

Although it has been reported in the literature that some combinations tested in our study provide an antibiofilm effect, there are very few studies explaining these mechanisms [26]. In the presented study, cellular metabolomes of *P. aeruginosa* treated with sub-MBIC concentrations of both ciprofloxacin and combinations of ciprofloxacin with curcumin, baicalein, cinnamaldehyde, and esculetin hydrate were analyzed to clarify the mechanisms of action for the compounds. We observed that uracil (−7.7 and −5.9-fold in combination with curcumin and cinnamaldehyde, respectively) and UMP metabolites (−50-fold in combination with cinnamaldehyde) involved in pyrimidine synthesis were significantly reduced in cells treated with curcumin and cinnamaldehyde combinations compared with ciprofloxacin alone. Pyrimidine synthesis is a complex pathway that begins with the transformation of L-glutamate to uridine monophosphate (UMP) for RNA and DNA production [27,28]. Pyrimidine synthesis plays a significant role in the regulation of many cell functions of *P. aeruginosa* [29]. In phenotypic studies, uracil is found to be effective on elastase, rhamnolipid synthesis, pseudomonas quinolone signal and swarming action, which are among the QS-related virulence factors. Whole transcriptome analysis of the UMP synthesis changed mutant strains showed that hundreds of QS-related genes and biofilm formation were suppressed and inhibited. However, the addition of uracil to the medium provided the re-expression of these genes and biofilm formation [30].

The down-regulated QS gene results obtained for curcumin and cinnamaldehyde, which provide a synergistic effect to ciprofloxacin in killing biofilm cells, and decreased uracil and UMP levels, support studies expressing the link between pyrimidine synthesis and QS inhibition in *P. aeruginosa*. In contrast to curcumin and cinnamaldehyde, the uracil and UMP metabolite levels and the upregulation of all genes increased in biofilm cells treated with combinations of ciprofloxacin with baicalein and esculin hydrate. These results show that one of the multifactorial mechanisms that can cause QS inhibition by curcumin and cinnamaldehyde is the uracil and UMP mediated pyrimidine mechanism.

Alginate is a capsular polysaccharide that has a significant role in *P. aeruginosa* virulence by helping recover from the effects of antipseudomonal drugs and the host immune system [31]. Ahmar et al. (2019) demonstrated a direct relationship between the UMP and UTP pyrimidine concentration and the mucoidity of *P. aeruginosa* at the level of transcriptional regulation mediated by the *PalgD* activity [27]. Similarly, we found the decreased uracil and UMP concentration and alginate gene expression levels in cells exposed to combinations of ciprofloxacin with curcumin and cinnamaldehyde, whereas increased levels of uracil and UMP concentration and gene expression in cells exposed to combinations of ciprofloxacin with baicalein and esculin hydrate were found. Therefore, another possible mechanism of killing biofilm cells with curcumin and cinnamaldehyde may be the decrement in alginate synthesis, which is indirectly associated with the decrease in pyrimidine metabolite levels.

Lipopolysaccharide (LPS) is a complex glycolipid composed of lipid A, core o-saccharide and hypervariable long-chain o-polysaccharide and plays important roles in the integrity of the outer membrane permeability barrier of *P. aeruginosa* [32]. Phosphoglycerate (−25-fold), cytidine-monophosphate (−2.85-fold), glycine (−2.5-fold), and ribulose-5-phosphate (−4.3-fold) metabolites, detected in lower amounts in cells exposed to a combination of ciprofloxacin with curcumin, play an important role in different stages of LPS synthesis.

Biogenic amines (BAs) regulate chromosomal and ribosomal organization; DNA, RNA, and protein synthesis in bacteria; and protect bacteria from osmotic stress. They are also known to regulate the swarming action and biofilm formation of *P. aeruginosa* [33,34]. Unlike those exposed to combinations of ciprofloxacin with baicalin and esculin hydrate, cells treated with combinations of ciprofloxacin with curcumin and cinnamaldehyde had a low level of 2-phenyl-ethylamine, 2-amino-1-phenyl ethanol, and tyramine. The decrease in BA levels in the cells treated with combinations with curcumin and cinnamaldehyde could be another reason for *P. aeruginosa* biofilm inhibition.

In conclusion, our multifactorial results, such as uracil and UMP-mediated pyrimidine synthesis, LPS biosynthesis, alginate production, and QS systems, indicate the reasons for curcumin and cinnamaldehyde-mediated *P. aeruginosa* biofilm inhibition. Curcumin, cinnamaldehyde, baicalein, and esculin reduced the inhibitory concentration of ciprofloxacin against biofilm cells by up to 64-fold. Using these compounds together with ciprofloxacin will help achieve successful results in the fight against ciprofloxacin-resistant *P. aeruginosa* biofilm infections.

## Figures and Tables

**Table 1:** MICs, MBICs, and MBECs of the compounds and ciprofloxacin against *P. aeruginosa* PAO1.

Agents	MICs (mg/L)	MBICs (mg/L)		MBECs (mg/L)	
	Alone *	Alone **	Combinations *** (CIP + Compounds)	Alone **	Combinations *** (CIP + Compounds)
Ciprofloxacin	0.039	0.06	-	0.13	-
Curcumin	0.25	0.82	0.001 + 0.008	>0.82	0.13 + 2
Azathioprine	0.25	0.82	0.008 + 0.06	>0.82	0.13 + 2
Resveratrol	0.25	0.2	0.03 + 0.25	>0.82	0.13 + 2
Catechin hydrate	0.51	0.82	0.03 + 0.26	>0.82	>0.13 + 2
Baicalein	0.13	0.2	0.001 + 0.008	>0.82	0.13 + 2
l-canavanine	0.1	0.82	0.004 + 0.03	>0.82	0.13 + 2
4-nitropyridine N-oxide	0.02	0.41	0.008 + 0.06	>0.82	0.13 + 2
p-benzoquinone	0.06	0.41	0.008 + 0.06	>0.82	0.13 + 2
Esculetin hydrate	0.51	0.82	0.002 + 0.02	>0.82	0.13 + 2
Cinnamaldehyde	0.26	0.41	0.002 + 0.02	>0.82	0.13 + 2

MICs—minimum inhibitory concentrations; MBICs—minimum biofilm inhibitory concentrations; MBECs—minimum biofilm eradication concentrations; mg/L—milligram per liter. * Minimum inhibition concentrations at which antimicrobial agents alone act against *P. aeruginosa* planktonic cells. ** Minimum biofilm inhibition concentrations at which antimicrobial agents alone act against *P. aeruginosa* biofilm cells. *** Minimum biofilm eradication concentrations at which compounds combined with ciprofloxacin act against *P. aeruginosa* biofilm cells.

**Table 2:** Comparison of metabolic profiling of *P. aeruginosa* biofilm cells treated with ciprofloxacin alone and combinations of ciprofloxacin with curcumin and cinnamaldehyde. The biofilm cells were grown in brain heart infusion agar (BHI) broth at 37 °C for 24 h, followed by further treatment with antimicrobial agents for another 24 h. The results are shown as the average.

		*Curc.*	*Cinnam.*			*Curc.*	*Cinnam.*
Carbohydrate	Citric acid	0.44↓	-	Nitrogen	Pyroglutamic acid	0.13↓	1.7↑
	Lactose	0.32↓	-		Aspartic acid	0.03↓	2.08↑
	Glucose	4.04↑	-		Glutamic acid	0.06↓	-
	Melibiose	0.1↓	1.83↑		N-acetyl-l-glutamic acid	0.3↓	-
	Mannose	1.78↑	-				
	Maltotriose	0.02↓	2.92↑	Lipid	Glyceric acid	0.25↓	-
	Maltotriitol	0.01↓	2.61↑		Glycine	0.4↓	-
	Melezitose	0.01↓	2.3↑		Glycerol-phosphate	0.35↓	-
	Glucuronic acid	0.24↓	-		Beta-glycerolphosphate	0.42↓	-
Amino Acid	Threonine	0.43↓	-				
					Glyceraldehyde	1.83↑	-
	Proline	0.24↓	-		3-phosphoglycerate	0.04↓	-
	Methionine	0.28↓	-				
	Lysine	0.01↓	0.12↓	Nucleotid	Uracil	0.13↓	0.17↓
	Phenylalanine	0.49↓	-		Uridine 5’-monophosphate	1.5↑	0.02↓
	Tryptophane		0.19↓		Ribulose-5-phosphate	0.23↓	0.06↓
Amines	Phenylethylamine	0.03↓	0.43↓				
					Pyrophosphate	6.58↑	-
	2-amino-1-phenylethanol	0.01↓	0.42↓		Cytidine-5’-monophosphate	0.35↓	-
	Tyramine	0.14↓	-		Porphine	3.19↑	-

**Table 3:** Comparison of metabolic profiling of *P. aeruginosa* biofilm cells treated with ciprofloxacin alone and combinations of ciprofloxacin with baicalein and esculetin hydrate. The biofilm cells were grown in BHI broth at 37 °C for 24 h, followed by further treatment with antimicrobial agents for another 24 h. The results are shown as the average.

		*Esculetin hyd*	*Baicalein hyd*
Carbohydrate	Fumaric acid	-	1.61↑
	Malic acid	-	1.64↑
	Melibiose	1.51↑	1.71↑
	Mannose	1.47↑	1.83↑
	Maltotriose	2.08↑	-
	Maltotriitol	1.75↑	-
	Melezitose	1.56↑	-
	Glucose-6-phosphate	-	3.15↑
Amino Acid	Aspartic Acid	2.8↑	-
	Lysine	4.13↑	-
	Tryptophane	1.87↑	2.89↑
Nitrogen	Pyroglutamic Acid	1.65↑	-
	Glutamic acid	2.37↑	-
Amines	Phenylethylamine	3.52↑	4.39↑
	2-amino-1-phenylethanol	12.83↑	1.47↑
	Tyramine	1.68↑	2.51↑
Lipid	Glycerol-phosphate	-	1.79↑
	Beta-glycerolphosphate	-	1.65↑
Nucleotid	Uracil	6.11↑	11.01↑
	Uridine 5’-monophosphate	0.41↓	3.81↑
	Ribulose-5-phosphate	1.38↑	9.27↑
	Pyrophosphate	1.49↑	5.89↑

## Data Availability

The data generated is contained within the article.

## Supplementary Materials

The following are available online at https://www.mdpi.com/article/10.3390/antibiotics10040414/s1, Table S1. Metabolites and relative amounts identified by GC-MS analysis. Metabolites that differ between groups are written in bold. *: *p* < 0.05 indicates that the difference is statistically significant. **: Data are values after normalization MSTUS and mean centering. Table S2: Primers for *P. aeruginosa* for RT-qPCR. (* reference genes), Figure S1. Scanning electron micrographs of single (*P. aeruginosa*) and triple-species biofilms formed by *P. aeruginosa*, *S. aureus* and *E. faecalis*. Panel A: *P. aeruginosa*, magnification 5.000×, Panel B: *P. aeruginosa*, magnification 20.000×, Panel C: Triple-species biofilm, magnification 5.000×, Panel D: Triple-species biofilm, magnification 20.000×. *S.a*: *Staphylococcus aureus*, *P.a*: *Pseudomonas aeruginosa*, *E.f*: *Enterococcus faecalis*. Figure S2: Principal component analysis (PCA) score plots of metabolic profiles in *P. aeruginosa* biofilms. Cip: Ciprofloxacin, Cip + Bai: Ciprofloxacin and Baicalein, Cip + Cur: Ciprofloxacin and Curcumin, Cip + Esc: Ciprofloxacin and Esculetin hydrate, Cip + Cal: Ciprofloxacin and Cinnamaldehyde.
