# Supplementary figures and images for: Sub-Inhibitory Concentrations of Ciprofloxacin Alone and Combinations with Plant-Derived Compounds against P. aeruginosa Biofilms and Their Effects on the Metabolomic Profile of P. aeruginosa Biofilms
Source: Antibiotics (Basel). 2021 Apr 9;10(4):414. doi: 10.3390/antibiotics10040414 (PMC8070142; doi:10.3390/antibiotics10040414)

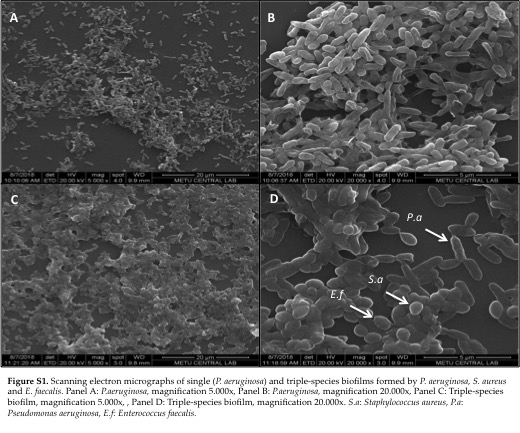

Supplement: Supplementary file 1 [file antibiotics-10-00414-s001.zip › Supplementary files/Supp Figure 1.jpg]

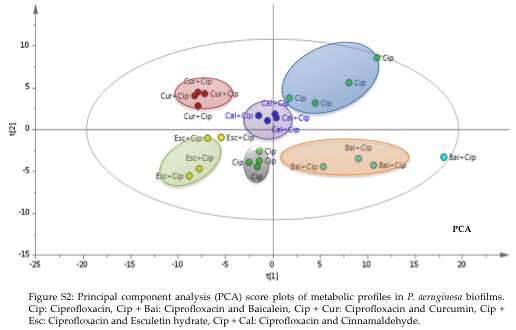

Supplement: Supplementary file 1 [file antibiotics-10-00414-s001.zip › Supplementary files/Supp Figure 2.jpg]
