# Supplementary material for: Sub-Inhibitory Concentrations of Ciprofloxacin Alone and Combinations with Plant-Derived Compounds against P. aeruginosa Biofilms and Their Effects on the Metabolomic Profile of P. aeruginosa Biofilms
Source: Antibiotics (Basel). 2021 Apr 9;10(4):414. doi: 10.3390/antibiotics10040414 (PMC8070142; doi:10.3390/antibiotics10040414)
Supplement: Supplementary file 1 [file antibiotics-10-00414-s001.zip › Supplementary files/Supplement Table 1.doc]

**Table S1.** Metabolites and relative amounts identified by GC-MS analysis.

| **Metabolite name** | **CIP** | | | **CAL+CIP** | | | | **CUR+CIP** | | | **ESC+CIP** | | | **BAI+CIP** | | | **P value** | | | |
| --- | --- | --- | --- | --- | --- | --- | --- | --- | --- | --- | --- | --- | --- | --- | --- | --- | --- | --- | --- | --- |
|  | Mean±SE | | | Mean±SE | | | | Mean±SE | | | Mean±SE | | | Mean±SE | | | CIP-(CUR+CIP) | CIP-(BAI+CIP) | CIP-(CAL+CIP) | CIP-(ESC+CIP) |
| 1,2-cyclohexanedione | 1,602 | ± | 0,115 | | 1,721 | ± | 0,086 | 0,455 | ± | 0,019 | 0,659 | ± | 0,088 | 2,415 | ± | 0,170 | **0,000** | **0,014** | 0,502 | **0,001** |
| 1,3-diaminopropane | 1,653 | ± | 0,140 | | 1,416 | ± | 0,064 | 1,386 | ± | 0,062 | 0,776 | ± | 0,049 | 0,889 | ± | 0,090 | 0,183 | **0,007** | 0,233 | **0,002** |
| 2-amino-1-phenylethanol | 2,731 | ± | 0,678 | | 0,038 | ± | 0,018 | 0,016 | ± | 0,006 | 0,010 | ± | 0,004 | 4,013 | ± | 0,913 | **0,013** | 0,367 | **0,014** | **0,013** |
| 2-ketobutyric acid | 1,110 | ± | 0,062 | | 1,316 | ± | 0,025 | 1,400 | ± | 0,059 | 1,071 | ± | 0,072 | 1,225 | ± | 0,069 | **0,026** | 0,326 | **0,036** | 0,734 |
| 2-keto-l-gulonic acid | 1,135 | ± | 0,104 | | 0,400 | ± | 0,038 | 0,312 | ± | 0,028 | 0,530 | ± | 0,072 | 3,930 | ± | 0,358 | **0,001** | **0,001** | **0,001** | **0,006** |
| 2-phosphoglyceric acid | 1,794 | ± | 0,111 | | 1,406 | ± | 0,047 | 0,944 | ± | 0,014 | 0,763 | ± | 0,062 | 1,432 | ± | 0,099 | **0,001** | 0,079 | **0,032** | **0,000** |
| 3-methyl-2-oxobutanoic acid | 1,246 | ± | 0,078 | | 1,164 | ± | 0,027 | 1,208 | ± | 0,053 | 0,986 | ± | 0,052 | 0,884 | ± | 0,182 | 0,735 | 0,165 | 0,425 | 0,054 |
| 3-phosphoglycerate | 2,820 | ± | 0,157 | | 0,995 | ± | 0,030 | 0,104 | ± | 0,007 | 0,403 | ± | 0,044 | 1,995 | ± | 0,125 | **0,000** | **0,012** | **0,000** | **0,000** |
| 4-hydroxyphenylacetic acid | 0,786 | ± | 0,064 | | 0,828 | ± | 0,038 | 0,783 | ± | 0,100 | 0,679 | ± | 0,161 | 1,089 | ± | 0,067 | 0,985 | **0,030** | 0,640 | 0,612 |
| 5-aminovaleric acid | 1,251 | ± | 0,110 | | 1,127 | ± | 0,049 | 0,509 | ± | 0,048 | 0,590 | ± | 0,077 | 2,552 | ± | 0,229 | **0,002** | **0,004** | 0,410 | **0,005** |
| 6-deoxy-glucose | 1,134 | ± | 0,202 | | 0,878 | ± | 0,041 | 0,950 | ± | 0,039 | 0,732 | ± | 0,054 | 1,049 | ± | 0,073 | 0,468 | 0,742 | 0,323 | 0,147 |
| Adenosine-5-monophosphate | 1,410 | ± | 0,164 | | 1,729 | ± | 0,072 | 0,526 | ± | 0,009 | 0,687 | ± | 0,094 | 2,350 | ± | 0,311 | **0,004** | 0,060 | 0,175 | **0,016** |
| Adipic acid | 1,239 | ± | 0,118 | | 1,180 | ± | 0,027 | 1,160 | ± | 0,036 | 0,642 | ± | 0,064 | 0,835 | ± | 0,037 | 0,596 | **0,030** | 0,687 | **0,008** |
| Alanine | 1,469 | ± | 0,109 | | 0,990 | ± | 0,038 | 0,754 | ± | 0,042 | 0,736 | ± | 0,055 | 1,414 | ± | 0,089 | **0,002** | 0,745 | **0,011** | **0,002** |
| Allo-inositol | 1,301 | ± | 0,096 | | 1,243 | ± | 0,194 | 1,438 | ± | 0,309 | 0,714 | ± | 0,073 | 1,295 | ± | 0,087 | 0,728 | 0,969 | 0,822 | **0,006** |
| Allothreonine | 0,909 | ± | 0,080 | | 1,449 | ± | 0,041 | 0,866 | ± | 0,046 | 0,791 | ± | 0,086 | 1,204 | ± | 0,091 | 0,704 | 0,079 | **0,002** | 0,419 |
| Alpha ketoglutaric acid | 1,472 | ± | 0,112 | | 1,070 | ± | 0,021 | 0,710 | ± | 0,044 | 0,481 | ± | 0,039 | 1,557 | ± | 0,139 | **0,002** | 0,693 | **0,022** | **0,000** |
| Alpha-glucosamine-phosphate | 1,056 | ± | 0,072 | | 1,284 | ± | 0,037 | 1,010 | ± | 0,085 | 1,219 | ± | 0,105 | 1,716 | ± | 0,752 | 0,730 | 0,478 | 0,051 | 0,309 |
| Arachidic acid | 1,275 | ± | 0,081 | | 1,280 | ± | 0,090 | 1,150 | ± | 0,065 | 0,857 | ± | 0,097 | 0,765 | ± | 0,027 | 0,336 | **0,002** | 0,977 | **0,029** |
| Aspartic acid | 3,320 | ± | 0,387 | | 0,712 | ± | 0,075 | 0,097 | ± | 0,015 | 0,046 | ± | 0,009 | 2,979 | ± | 0,358 | **0,000** | 0,596 | **0,001** | **0,000** |
| Beta-glycerolphosphate | 1,278 | ± | 0,092 | | 1,071 | ± | 0,030 | 0,532 | ± | 0,023 | 0,647 | ± | 0,065 | 2,112 | ± | 0,150 | **0,001** | **0,006** | 0,114 | **0,003** |
| Cholestrol | 0,322 | ± | 0,211 | | 0,100 | ± | 0,014 | 1,799 | ± | 1,435 | 0,141 | ± | 0,013 | 0,130 | ± | 0,009 | 0,412 | 0,460 | 0,397 | 0,487 |
| Citric acid | 2,123 | ± | 0,146 | | 1,134 | ± | 0,024 | 0,927 | ± | 0,041 | 0,572 | ± | 0,096 | 1,535 | ± | 0,141 | **0,000** | **0,046** | **0,001** | **0,000** |
| Cytindine-5'-monophosphate | 1,804 | ± | 0,506 | | 0,691 | ± | 0,038 | 0,632 | ± | 0,013 | 0,617 | ± | 0,055 | 1,072 | ± | 0,037 | 0,091 | 0,258 | 0,106 | 0,090 |
| Daidzein | 0,926 | ± | 0,044 | | 0,790 | ± | 0,017 | 1,122 | ± | 0,034 | 0,923 | ± | 0,068 | 0,872 | ± | 0,026 | **0,022** | 0,398 | **0,047** | 0,976 |
| Dl-glyceraldehyde | 0,868 | ± | 0,115 | | 1,060 | ± | 0,057 | 1,587 | ± | 0,084 | 1,142 | ± | 0,088 | 0,611 | ± | 0,112 | **0,005** | 0,214 | 0,244 | 0,153 |
| Dl-isoleucine | 1,462 | ± | 0,106 | | 1,069 | ± | 0,028 | 0,911 | ± | 0,054 | 0,887 | ± | 0,069 | 1,383 | ± | 0,086 | **0,007** | 0,635 | **0,021** | **0,008** |
| Estrone | 0,813 | ± | 0,034 | | 0,761 | ± | 0,011 | 0,957 | ± | 0,038 | 0,638 | ± | 0,035 | 0,651 | ± | 0,025 | 0,051 | **0,016** | 0,252 | **0,021** |
| Fumaric acid | 1,291 | ± | 0,096 | | 1,067 | ± | 0,047 | 0,728 | ± | 0,022 | 0,716 | ± | 0,071 | 2,080 | ± | 0,168 | **0,003** | **0,012** | 0,118 | **0,006** |
| Galactinol | 1,596 | ± | 0,270 | | 1,934 | ± | 0,066 | 0,163 | ± | 0,019 | 0,393 | ± | 0,033 | 2,799 | ± | 0,272 | **0,004** | **0,035** | 0,333 | **0,009** |
| Glucoheptonic acid | 1,414 | ± | 0,118 | | 1,646 | ± | 0,181 | 0,646 | ± | 0,041 | 0,834 | ± | 0,162 | 2,145 | ± | 0,154 | **0,002** | **0,017** | 0,389 | **0,046** |
| Gluconic acid | 1,311 | ± | 0,105 | | 0,385 | ± | 0,035 | 0,407 | ± | 0,037 | 0,674 | ± | 0,110 | 4,462 | ± | 0,372 | **0,000** | **0,000** | **0,000** | **0,011** |
| Glucose | 0,414 | ± | 0,050 | | 0,580 | ± | 0,012 | 1,671 | ± | 0,013 | 1,433 | ± | 0,128 | 0,336 | ± | 0,026 | **0,000** | 0,278 | **0,031** | **0,001** |
| Glucose-6-phosphate | 0,773 | ± | 0,112 | | 0,493 | ± | 0,068 | 0,746 | ± | 0,020 | 0,980 | ± | 0,132 | 2,432 | ± | 0,227 | 0,849 | **0,001** | 0,114 | 0,339 |
| Glucuronic acid | 2,634 | ± | 0,231 | | 1,170 | ± | 0,054 | 0,640 | ± | 0,092 | 0,617 | ± | 0,069 | 1,485 | ± | 0,109 | **0,000** | **0,008** | **0,002** | **0,000** |
| Glutamic acid | 3,258 | ± | 0,513 | | 0,486 | ± | 0,127 | 0,183 | ± | 0,038 | 0,066 | ± | 0,032 | 3,109 | ± | 0,515 | **0,002** | 0,865 | **0,004** | **0,002** |
| Glyceric acid | 1,573 | ± | 0,117 | | 0,700 | ± | 0,024 | 0,389 | ± | 0,016 | 0,323 | ± | 0,021 | 2,507 | ± | 0,212 | **0,000** | **0,016** | **0,001** | **0,000** |
| Glycerol | 0,900 | ± | 0,009 | | 1,147 | ± | 0,017 | 1,405 | ± | 0,049 | 0,912 | ± | 0,066 | 0,803 | ± | 0,016 | **0,000** | **0,004** | **0,000** | 0,886 |
| Glycerol-phosphate | 1,324 | ± | 0,109 | | 1,003 | ± | 0,037 | 0,462 | ± | 0,015 | 0,607 | ± | 0,061 | 2,369 | ± | 0,156 | **0,000** | **0,003** | 0,052 | **0,002** |
| Glycine | 1,638 | ± | 0,120 | | 1,094 | ± | 0,053 | 0,657 | ± | 0,030 | 0,668 | ± | 0,055 | 1,927 | ± | 0,139 | **0,000** | 0,223 | **0,012** | **0,001** |
| Glycolic acid | 0,996 | ± | 0,075 | | 1,059 | ± | 0,033 | 1,205 | ± | 0,027 | 0,894 | ± | 0,058 | 0,904 | ± | 0,046 | 0,063 | 0,399 | 0,529 | 0,386 |
| Heptadecanoic acid | 1,321 | ± | 0,077 | | 1,199 | ± | 0,034 | 0,908 | ± | 0,038 | 0,693 | ± | 0,068 | 1,276 | ± | 0,082 | **0,006** | 0,740 | 0,252 | **0,002** |
| Inosine | 2,217 | ± | 0,363 | | 0,829 | ± | 0,047 | 0,675 | ± | 0,049 | 0,457 | ± | 0,031 | 1,687 | ± | 0,220 | **0,011** | 0,322 | **0,017** | **0,006** |
| Isoleucine | 1,449 | ± | 0,152 | | 0,994 | ± | 0,034 | 0,923 | ± | 0,037 | 0,930 | ± | 0,073 | 1,544 | ± | 0,122 | **0,027** | 0,690 | **0,045** | **0,037** |
| Isopropyl beta-1-thiogalactopyranoside | 2,435 | ± | 1,207 | | 1,114 | ± | 0,080 | 0,634 | ± | 0,018 | 0,640 | ± | 0,077 | 1,229 | ± | 0,085 | 0,244 | 0,421 | 0,381 | 0,246 |
| Lactic acid | 1,217 | ± | 0,075 | | 1,046 | ± | 0,073 | 0,696 | ± | 0,085 | 0,724 | ± | 0,069 | 1,721 | ± | 0,149 | **0,007** | **0,040** | 0,206 | **0,006** |
| Lactobionic acid | 1,313 | ± | 0,089 | | 1,228 | ± | 0,036 | 1,267 | ± | 0,062 | 0,847 | ± | 0,078 | 1,091 | ± | 0,059 | 0,728 | 0,121 | 0,475 | **0,014** |
| Lactose | 1,655 | ± | 0,187 | | 1,675 | ± | 0,047 | 0,527 | ± | 0,014 | 0,770 | ± | 0,088 | 2,092 | ± | 0,278 | **0,002** | 0,302 | 0,931 | **0,010** |
| Lauric acid | 1,360 | ± | 0,036 | | 1,570 | ± | 0,143 | 0,646 | ± | 0,032 | 0,839 | ± | 0,135 | 1,910 | ± | 0,050 | **0,000** | **0,000** | 0,266 | **0,018** |
| Leucine | 1,472 | ± | 0,152 | | 1,001 | ± | 0,033 | 0,970 | ± | 0,034 | 0,949 | ± | 0,068 | 1,482 | ± | 0,132 | **0,031** | 0,965 | **0,039** | **0,034** |
| Linoleic acid | 1,388 | ± | 0,019 | | 1,108 | ± | 0,027 | 0,928 | ± | 0,027 | 0,792 | ± | 0,080 | 1,215 | ± | 0,068 | **0,000** | 0,079 | **0,000** | **0,001** |
| Lysine | 2,820 | ± | 0,601 | | 0,029 | ± | 0,018 | 0,029 | ± | 0,012 | 0,014 | ± | 0,009 | 3,423 | ± | 0,587 | **0,007** | 0,557 | **0,007** | **0,007** |
| Lyxose | 0,662 | ± | 0,077 | | 0,451 | ± | 0,027 | 0,821 | ± | 0,011 | 1,314 | ± | 0,167 | 2,327 | ± | 0,231 | 0,128 | **0,001** | 0,066 | **0,022** |
| Malic acid | 1,217 | ± | 0,077 | | 1,058 | ± | 0,034 | 0,715 | ± | 0,026 | 0,502 | ± | 0,046 | 1,995 | ± | 0,165 | **0,002** | **0,010** | 0,153 | **0,000** |
| Maltotriitol | 3,396 | ± | 0,511 | | 1,235 | ± | 0,089 | 0,034 | ± | 0,004 | 0,341 | ± | 0,036 | 2,310 | ± | 0,454 | **0,001** | 0,218 | **0,011** | **0,002** |
| Maltotriose | 2,632 | ± | 0,404 | | 1,606 | ± | 0,136 | 0,048 | ± | 0,003 | 0,293 | ± | 0,028 | 2,607 | ± | 0,368 | **0,001** | 0,969 | 0,082 | **0,002** |
| Mannitol | 1,850 | ± | 0,130 | | 1,289 | ± | 0,022 | 1,012 | ± | 0,037 | 0,772 | ± | 0,050 | 0,840 | ± | 0,041 | **0,002** | **0,001** | **0,010** | **0,001** |
| Mannose | 0,708 | ± | 0,033 | | 0,776 | ± | 0,034 | 1,260 | ± | 0,030 | 1,288 | ± | 0,116 | 1,295 | ± | 0,082 | **0,000** | **0,001** | 0,255 | **0,006** |
| Melezitose | 3,078 | ± | 0,566 | | 1,321 | ± | 0,071 | 0,032 | ± | 0,001 | 0,431 | ± | 0,050 | 2,274 | ± | 0,460 | **0,003** | 0,376 | **0,037** | **0,007** |
| Melibiose | 1,563 | ± | 0,228 | | 1,990 | ± | 0,052 | 0,153 | ± | 0,022 | 0,477 | ± | 0,051 | 2,673 | ± | 0,307 | **0,002** | **0,046** | 0,164 | **0,007** |
| Methionine | 1,826 | ± | 0,136 | | 0,874 | ± | 0,043 | 0,519 | ± | 0,011 | 0,548 | ± | 0,067 | 2,260 | ± | 0,295 | **0,000** | 0,291 | **0,001** | **0,000** |
| Methyl oleate | 1,702 | ± | 0,033 | | 1,442 | ± | 0,116 | 0,660 | ± | 0,029 | 0,950 | ± | 0,116 | 1,967 | ± | 0,143 | **0,000** | 0,169 | 0,111 | **0,002** |
| Methyl palmitate | 1,429 | ± | 0,042 | | 1,215 | ± | 0,066 | 1,564 | ± | 0,024 | 1,103 | ± | 0,098 | 1,315 | ± | 0,033 | **0,050** | 0,113 | 0,055 | **0,038** |
| Methyl stearate | 1,233 | ± | 0,044 | | 0,985 | ± | 0,058 | 1,980 | ± | 0,027 | 1,291 | ± | 0,125 | 1,071 | ± | 0,024 | **0,000** | **0,031** | **0,025** | 0,717 |
| Methyl-beta-galactopyranoside | 0,772 | ± | 0,041 | | 0,857 | ± | 0,026 | 0,998 | ± | 0,026 | 0,806 | ± | 0,070 | 0,802 | ± | 0,050 | **0,007** | 0,692 | 0,178 | 0,726 |
| Myo-inositol | 1,408 | ± | 0,136 | | 1,129 | ± | 0,046 | 0,970 | ± | 0,056 | 0,816 | ± | 0,061 | 1,538 | ± | 0,110 | **0,042** | 0,545 | 0,144 | **0,014** |
| Myristic acid | 1,290 | ± | 0,039 | | 1,166 | ± | 0,035 | 0,863 | ± | 0,025 | 0,643 | ± | 0,057 | 1,271 | ± | 0,053 | **0,000** | 0,808 | 0,088 | **0,000** |
| N-acetyl-l-glutamic acid | 1,835 | ± | 0,065 | | 1,280 | ± | 0,028 | 0,546 | ± | 0,038 | 0,799 | ± | 0,062 | 1,710 | ± | 0,161 | **0,000** | 0,554 | **0,000** | **0,000** |
| Naringin | 1,190 | ± | 0,165 | | 1,743 | ± | 0,102 | 0,859 | ± | 0,027 | 0,971 | ± | 0,139 | 1,998 | ± | 0,238 | 0,137 | 0,052 | **0,048** | 0,413 |
| N-ethylglycine | 1,314 | ± | 0,206 | | 1,412 | ± | 0,057 | 0,371 | ± | 0,008 | 0,511 | ± | 0,038 | 2,613 | ± | 0,162 | **0,007** | **0,005** | 0,707 | **0,016** |
| Oleic acid | 1,784 | ± | 0,122 | | 1,326 | ± | 0,034 | 0,494 | ± | 0,007 | 0,511 | ± | 0,048 | 2,438 | ± | 0,155 | **0,000** | **0,028** | **0,020** | **0,000** |
| O-phosphocolamine | 2,267 | ± | 0,151 | | 1,109 | ± | 0,091 | 0,534 | ± | 0,048 | 0,405 | ± | 0,116 | 2,369 | ± | 0,113 | **0,000** | 0,655 | **0,001** | **0,000** |
| Oxalacetic acid | 1,249 | ± | 0,112 | | 0,965 | ± | 0,051 | 0,928 | ± | 0,076 | 0,844 | ± | 0,056 | 1,239 | ± | 0,052 | 0,086 | 0,946 | 0,094 | **0,031** |
| Oxalic acid | 0,888 | ± | 0,051 | | 1,052 | ± | 0,020 | 1,110 | ± | 0,050 | 0,857 | ± | 0,055 | 0,971 | ± | 0,059 | **0,036** | 0,391 | **0,042** | 0,730 |
| Palatinose | 1,408 | ± | 0,170 | | 1,807 | ± | 0,081 | 1,035 | ± | 0,173 | 1,314 | ± | 0,128 | 0,919 | ± | 0,067 | 0,231 | 0,059 | 0,116 | 0,714 |
| Palmitic acid | 1,208 | ± | 0,019 | | 1,159 | ± | 0,021 | 0,957 | ± | 0,032 | 0,789 | ± | 0,072 | 1,270 | ± | 0,039 | **0,001** | 0,253 | 0,191 | **0,003** |
| Palmitoleic acid | 1,247 | ± | 0,025 | | 1,448 | ± | 0,078 | 0,776 | ± | 0,020 | 0,747 | ± | 0,090 | 1,741 | ± | 0,044 | **0,000** | **0,000** | 0,077 | **0,004** |
| Phenylalanine | 1,577 | ± | 0,131 | | 1,091 | ± | 0,040 | 0,767 | ± | 0,040 | 0,612 | ± | 0,083 | 2,006 | ± | 0,253 | **0,002** | 0,240 | **0,022** | **0,002** |
| Phenylethylamine | 1,144 | ± | 0,139 | | 0,307 | ± | 0,011 | 0,039 | ± | 0,003 | 0,066 | ± | 0,007 | 5,015 | ± | 0,536 | **0,000** | **0,001** | **0,002** | **0,001** |
| Phosphoric acid | 0,924 | ± | 0,014 | | 0,895 | ± | 0,005 | 1,114 | ± | 0,003 | 1,035 | ± | 0,088 | 0,897 | ± | 0,017 | **0,000** | 0,334 | 0,146 | 0,324 |
| Porphine | 0,456 | ± | 0,029 | | 0,549 | ± | 0,025 | 1,455 | ± | 0,043 | 0,495 | ± | 0,037 | 0,511 | ± | 0,036 | **0,000** | 0,343 | 0,082 | 0,503 |
| Proline | 1,919 | ± | 0,160 | | 0,894 | ± | 0,032 | 0,454 | ± | 0,034 | 0,540 | ± | 0,040 | 1,783 | ± | 0,059 | **0,000** | 0,517 | **0,002** | **0,000** |
| Putrescine | 0,594 | ± | 0,038 | | 1,045 | ± | 0,036 | 0,902 | ± | 0,071 | 1,048 | ± | 0,099 | 2,651 | ± | 0,121 | **0,016** | **0,000** | **0,000** | **0,010** |
| Pyroglutamic acid | 2,541 | ± | 0,165 | | 1,054 | ± | 0,072 | 0,326 | ± | 0,027 | 0,161 | ± | 0,019 | 2,480 | ± | 0,183 | **0,000** | 0,839 | **0,000** | **0,000** |
| Pyrophosphate | 0,224 | ± | 0,105 | | 1,665 | ± | 0,217 | 1,472 | ± | 0,076 | 0,425 | ± | 0,091 | 1,318 | ± | 0,164 | **0,000** | **0,003** | **0,002** | 0,257 |
| Pyruvic acid | 1,174 | ± | 0,162 | | 1,240 | ± | 0,093 | 1,498 | ± | 0,030 | 1,039 | ± | 0,114 | 0,977 | ± | 0,068 | 0,140 | 0,369 | 0,772 | 0,577 |
| Raffinose | 0,923 | ± | 0,051 | | 0,997 | ± | 0,030 | 1,538 | ± | 0,005 | 0,974 | ± | 0,097 | 0,845 | ± | 0,084 | **0,000** | 0,514 | 0,321 | 0,702 |
| Ribitol | 1,587 | ± | 0,136 | | 1,234 | ± | 0,046 | 1,059 | ± | 0,051 | 0,673 | ± | 0,059 | 1,286 | ± | 0,107 | **0,020** | 0,183 | 0,077 | **0,002** |
| Ribulose-5-phosphate | 0,447 | ± | 0,054 | | 0,164 | ± | 0,035 | 0,102 | ± | 0,013 | 0,168 | ± | 0,015 | 4,143 | ± | 0,512 | **0,002** | **0,001** | **0,009** | **0,005** |
| Sedoheptulose | 1,079 | ± | 0,058 | | 1,012 | ± | 0,026 | 1,071 | ± | 0,119 | 0,540 | ± | 0,050 | 0,942 | ± | 0,058 | 0,960 | 0,198 | 0,398 | **0,001** |
| Serine | 1,472 | ± | 0,102 | | 1,191 | ± | 0,054 | 0,921 | ± | 0,051 | 0,729 | ± | 0,060 | 1,198 | ± | 0,064 | **0,006** | 0,098 | 0,081 | **0,002** |
| Sorbitol | 0,742 | ± | 0,053 | | 0,971 | ± | 0,121 | 0,850 | ± | 0,109 | 1,284 | ± | 0,141 | 0,990 | ± | 0,152 | 0,471 | 0,231 | 0,183 | **0,021** |
| Sphingosine | 1,703 | ± | 0,233 | | 1,697 | ± | 0,060 | 0,324 | ± | 0,008 | 0,551 | ± | 0,070 | 2,598 | ± | 0,341 | **0,002** | 0,109 | 0,985 | **0,006** |
| Squalene | 0,588 | ± | 0,156 | | 0,242 | ± | 0,001 | 0,346 | ± | 0,071 | 0,223 | ± | 0,053 | 0,223 | ± | 0,067 | 0,268 | 0,113 | 0,104 | 0,104 |
| Stearic acid | 1,134 | ± | 0,038 | | 1,093 | ± | 0,049 | 1,093 | ± | 0,054 | 0,863 | ± | 0,075 | 0,874 | ± | 0,027 | 0,613 | **0,003** | 0,587 | **0,031** |
| Succinic acid | 1,263 | ± | 0,095 | | 1,222 | ± | 0,022 | 0,985 | ± | 0,050 | 0,654 | ± | 0,048 | 1,311 | ± | 0,088 | 0,065 | 0,762 | 0,725 | **0,003** |
| Sucrose | 1,008 | ± | 0,070 | | 1,005 | ± | 0,034 | 1,346 | ± | 0,060 | 0,902 | ± | 0,069 | 0,835 | ± | 0,070 | **0,019** | 0,181 | 0,980 | 0,385 |
| Tagatose | 1,045 | ± | 0,075 | | 1,004 | ± | 0,032 | 1,275 | ± | 0,055 | 0,988 | ± | 0,077 | 1,391 | ± | 0,091 | 0,075 | **0,044** | 0,682 | 0,664 |
| Threonine | 1,751 | ± | 0,112 | | 1,039 | ± | 0,038 | 0,749 | ± | 0,022 | 0,671 | ± | 0,062 | 1,602 | ± | 0,088 | **0,000** | 0,400 | **0,002** | **0,000** |
| Thymine | 1,236 | ± | 0,154 | | 1,260 | ± | 0,071 | 1,516 | ± | 0,058 | 1,070 | ± | 0,113 | 1,025 | ± | 0,057 | 0,192 | 0,309 | 0,907 | 0,481 |
| Trehalose | 1,225 | ± | 0,110 | | 1,141 | ± | 0,032 | 1,413 | ± | 0,062 | 1,157 | ± | 0,087 | 0,601 | ± | 0,062 | 0,243 | **0,005** | 0,552 | 0,691 |
| Tryptophane | 0,650 | ± | 0,062 | | 0,148 | ± | 0,048 | 0,901 | ± | 0,172 | 0,354 | ± | 0,107 | 1,883 | ± | 0,290 | 0,282 | **0,011** | **0,001** | 0,083 |
| Tyramine | 1,375 | ± | 0,131 | | 0,587 | ± | 0,022 | 0,186 | ± | 0,009 | 0,214 | ± | 0,030 | 3,453 | ± | 0,287 | **0,000** | **0,001** | **0,002** | **0,000** |
| Tyrosine | 1,362 | ± | 0,187 | | 0,654 | ± | 0,071 | 0,807 | ± | 0,042 | 0,854 | ± | 0,142 | 1,831 | ± | 0,213 | **0,046** | 0,202 | **0,022** | 0,110 |
| Uracil | 0,520 | ± | 0,102 | | 0,123 | ± | 0,015 | 0,068 | ± | 0,005 | 0,070 | ± | 0,008 | 5,724 | ± | 2,505 | **0,009** | 0,122 | **0,016** | **0,009** |
| Uridine 5'-monophosphate | 0,081 | ± | 0,020 | | 0,096 | ± | 0,006 | 0,121 | ± | 0,007 | 0,237 | ± | 0,147 | 0,307 | ± | 0,046 | 0,152 | **0,008** | 0,536 | 0,397 |
| Valine | 1,399 | ± | 0,108 | | 1,002 | ± | 0,028 | 0,929 | ± | 0,028 | 0,897 | ± | 0,063 | 1,561 | ± | 0,102 | **0,011** | 0,383 | **0,021** | **0,013** |

Metabolites that differ between groups are written in bold.

*: P <0.05 indicates that the difference is statistically significant.

**: Data are values after normalization MSTUS and mean centering.
