# Supplementary material for: Sub-Inhibitory Concentrations of Ciprofloxacin Alone and Combinations with Plant-Derived Compounds against P. aeruginosa Biofilms and Their Effects on the Metabolomic Profile of P. aeruginosa Biofilms
Source: Antibiotics (Basel). 2021 Apr 9;10(4):414. doi: 10.3390/antibiotics10040414 (PMC8070142; doi:10.3390/antibiotics10040414)
Supplement: Supplementary file 1 [file antibiotics-10-00414-s001.zip › Supplementary files/Supplement Table 2.doc]

**Table S2.** Primers for *P. aeruginosa* for RT-qPCR.

| **Genes** | **Forward** | **Reverse** |
| --- | --- | --- |
| ***alg*** | CTACCTTCTCGGCCAACCT | GTCAGGGTCCCTTTCATCTG |
| ***psl*** | CGCTCACGGTGATTATGTTC | TACATGAACAACAGCAGGCA |
| ***lasR*** | ACGCTCAAGTGGAAAATTGG | GTAGATGGACGGTTCCCAGA |
| ***lasI*** | CTACAGCCTGCAGAACGACA | ATCTGGGTCTTGGCATTGAG |
| ***rhlR*** | AGGAATGACGGAGGCTTTTT | CCCGTAGTTCTGCATCTGGT |
| ***proC**** | GGCGTATTTCTTCCTGCTGA | CCTGCTCCACTAGTGCTTCG |

(* reference genes)
